# NIR-Activated ICG-Loaded M2 Macrophage Exosomes Ameliorate Periodontitis via Targeting Infection Inflammation and Oxidative Stress

**DOI:** 10.34133/research.1207

**Published:** 2026-04-27

**Authors:** Xincong Li, Xin Fu, Tianyu Zhang, Xiaofan Cheng, Hai Zhuang, Zhilei Mao, Shoushan Bu

**Affiliations:** ^1^ Department of Stomatology, the First Affiliated Hospital of Nanjing Medical University, Nanjing 210029, P.R. China.; ^2^ Changzhou Maternity and Child Health Care Hospital, Changzhou Medical Center, Nanjing Medical University, Changzhou 213003, Jiangsu, P.R. China.

## Abstract

Periodontitis is a chronic bacterial inflammatory disease. M2 macrophage-derived exosomes (M2-exos) possess targeted immunomodulatory abilities, but their role in mitigating oxidative stress (key for periodontitis treatment) remains unclear. In this study, we engineered M2-exos loaded with indocyanine green (ICG) (ICG@M2-exos) for the treatment of periodontitis. The constructed ICG@M2-exos effectively facilitated macrophage reprogramming from the M1 to the M2 phenotype, thereby resolving chronic inflammation and enhancing periodontal tissue repair. Under near-infrared irradiation, ICG conferred potent antibacterial efficacy against *Porphyromonas gingivalis* (*P. gingivalis*). Simultaneously, the exosomes released from ICG@M2-exos mitigated oxidative stress and decreased the expression of proinflammatory factors in THP-1 cells through promoting M2 polarization. In a rat model of *P. gingivalis*-induced periodontitis, the sustained release of ICG@M2-exos markedly expedited periodontal bone regeneration, accompanied by elevated levels of anti-inflammatory cytokines. Collectively, ICG-engineered M2-exos represent a promising strategy for tackling inflammatory periodontal conditions. This study demonstrates the dual advantages of ICG@M2-exos in near-infrared-responsive antibacterial activity and immunomodulation that work synergistically, laying a solid foundation for future clinical applications.

## Introduction

Periodontitis is a chronic bacterial inflammatory disease that destroys tooth-supporting structures, with complex crosstalk between pathogens and the immune microenvironment posing a major barrier to periodontal tissue regeneration [1]. Current antibiotic-based therapies have limited use due to drug resistance and ineffective biofilm elimination, while the dysregulated osteoimmune microenvironment further hinders tissue repair [2–7]. Thus, there is an urgent need for integrated strategies combining antibacterial, anti-inflammatory, and proregenerative effects [8–12].

Photodynamic therapy offers a promising antibacterial alternative, using photosensitizers like indocyanine green (ICG) activated by near-infrared (NIR) light to generate reactive oxygen species (ROS) for broad-spectrum bactericidal action without inducing resistance [13–17]. The favorable photophysical properties of ICG together with the deep tissue penetration of NIR make this combination ideal for targeting periodontal pathogens such as *Porphyromonas gingivalis* (*P. gingivalis*) [18,19].

Macrophage polarization is central to the pathological process of periodontal inflammation. Proinflammatory M1 macrophages exacerbate tissue damage, while anti-inflammatory M2 macrophages promote tissue repair [20–25]. M2 macrophage-derived exosomes (M2-exos) possess inherent inflammatory tropism, enabling targeted delivery of immunomodulatory cargo to rebalance the microenvironment [26–30]. Hypoxic preconditioning further enhances their anti-inflammatory potential, making them a robust tool for reprogramming macrophage phenotypes.

**Fig. 1.**
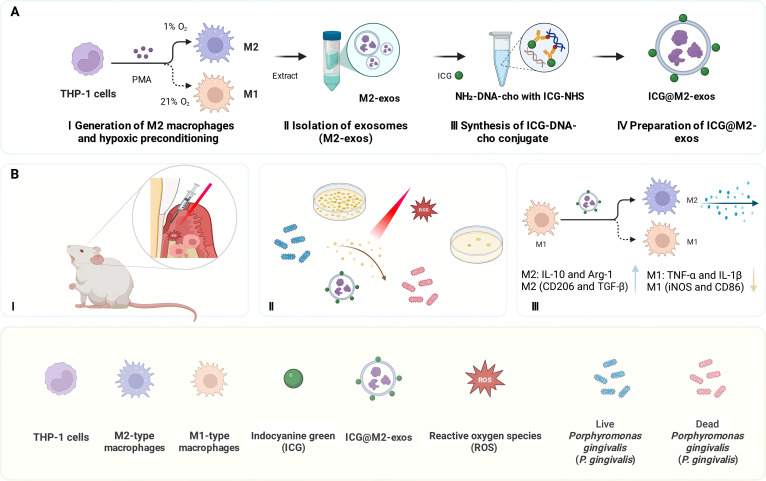
Schematic illustration of the design and therapeutic application of indocyanine green-loaded M2 macrophage-derived exosomes (ICG@M2-exos) for targeting periodontitis and modulating macrophage polarization. (A) Fabrication process of ICG@M2-exos: (I) THP-1 cells were differentiated into M2 macrophages under hypoxia and M1 macrophages under normoxia; (II) M2-exos were isolated from M2 macrophage supernatant; (III) ICG-DNA-cho conjugate was synthesized; (IV) ICG@M2-exos were prepared by loading the conjugate into M2-exos. (B) In vivo therapeutic mechanism: (I) Local administration of ICG@M2-exos to periodontal tissue; (II) near-infrared (NIR) irradiation-induced reactive oxygen species (ROS) generation for *Porphyromonas gingivalis* (*P. gingivalis*) elimination; (III) M2-exos mediated M1-to-M2 macrophage polarization, reducing inflammatory responses via modulation of macrophage marker expression. IL-10, interleukin-10; Arg-1, arginase-1; CD206, cluster of differentiation 206; TGF-β, transforming growth factor-β; TNF-α, tumor necrosis factor-α; IL-1β, interleukin-1β; iNOS, inducible nitric oxide synthase; CD86, cluster of differentiation 86; PMA, phorbol 12-myristate 13-acetate.

To address the multifaceted challenges in the pathogenesis of periodontitis, we engineered ICG-loaded M2-exos (ICG@M2-exos), a synergistic nanoplatform that integrates 2 core functionalities (Fig. 1A). First, under NIR irradiation, ICG mediates photodynamic bactericidal activity by generating ROS to irreversibly damage bacterial structures, thus effectively eliminating *P. gingivalis* and biofilms. Second, the exosomal platform significantly mitigates inflammation, a key driver of periodontal tissue destruction, by scavenging excessive ROS and restoring cellular redox balance (Fig. 1B). This multipronged design enables simultaneous targeting of infection, inflammation, and oxidative stress, creating a favorable microenvironment for periodontal bone regeneration and tissue repair. Collectively, this study offers a novel, biocompatible strategy for comprehensive periodontitis treatment with substantial potential in future clinical translation.

## Results and Discussion

### Preparation and characterization of ICG@M2-exos

**Fig. 2.**
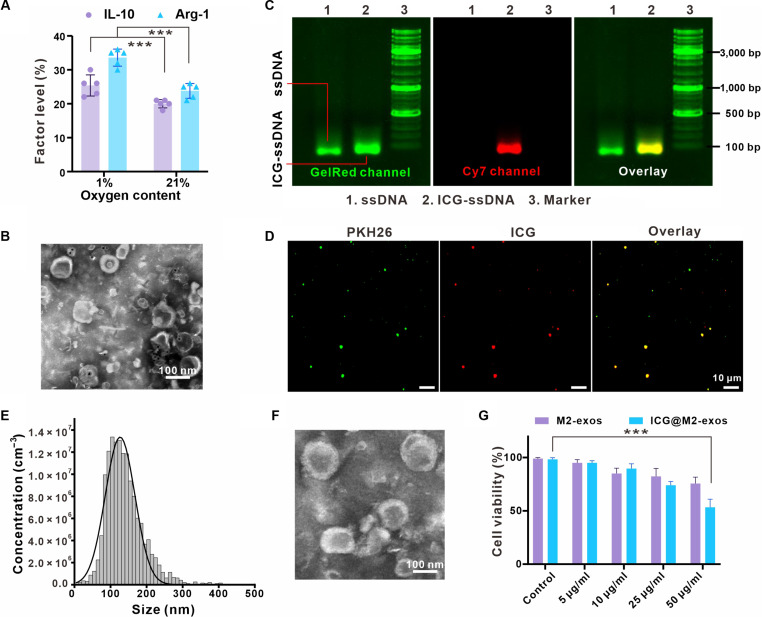
Preparation and characterization of M2 macrophage-derived exosomes (M2-exos) and indocyanine green (ICG)-labeled exosomes (ICG@M2-exos). (A) Expression levels of anti-inflammatory factors in exosomes derived from M2 macrophages compared to those from conventionally treated macrophages. ***P < 0.001. (B) Transmission electron microscopy (TEM) images of M2-exos (1% O_2_). (C) Agarose gel electrophoresis of cholesterol-modified DNA (cho-DNA) and the ICG-DNA-cho conjugate, visualized under Cy7 wavelength illumination to confirm the successful conjugation of ICG to DNA. (D) Confocal laser scanning microscope (CLSM) images demonstrating the colocalization of PKH26-labeled M2-exos (green) and ICG fluorescence (red). Scale bar: 10 μm. (E) Nanoparticle tracking analysis (NTA) revealing the size distribution of ICG@M2-exos, with an average diameter of 130.255 ± 7.79 nm. (F) TEM images showing the spherical, bilayer membrane structure of ICG@M2-exos. (G) Cell Counting Kit-8 (CCK-8) assay shows the excellent biocompatibility of both native M2-exos and ICG@M2-exos. Data are presented as means ± SD. ***P < 0.001. IL-10, interleukin-10; Arg-1, arginase-1; ssDNA, single-stranded DNA.

Macrophages play a pivotal role in maintaining immune homeostasis and regulating inflammatory responses [31]. M2-exos were isolated from the conditioned medium of THP-1-derived M2 macrophages via ultracentrifugation. THP-1 cells were first differentiated into M0 phenotype with phorbol 12-myristate 13-acetate (PMA) (24 h), followed by polarization to M2 type using either one of the following 2 protocols: (a) hypoxia pretreatment (1% O₂, 24 h); or (b) interleukin-4 (IL-4)/interleukin-13 (IL-13) stimulation (20 ng/ml each, 21% O₂, 24 h). Bicinchoninic acid assay showed that ~10 μg of exosomal protein was isolated from 10^6^ cells. As shown in Fig. 2A, M2-exos exhibited higher expression of anti-inflammatory factors compared to exosomes from conventionally cultured macrophages. Transmission electron microscopy (TEM) analysis confirmed the spherical morphology and structural integrity of M2-exos without aggregation (Fig. 2B).

ICG@M2-exos were then constructed by conjugating ICG to M2-exos surface via cholesterol (cho)-modified DNA anchor. Briefly, ICG-DNA-cho was synthesized through amide coupling between NH₂-DNA-cho and ICG-NHS, followed by insertion into exosomal membrane via hydrophobic interaction of cholesterol moiety (Fig. 2C). Colocalization assay with PKH26-labeled M2-exos verified successful ICG conjugation, as evidenced by the overlapping fluorescence signals in Cy7 channel (Fig. 2D). Agarose gel electrophoresis for the single-strand DNA further confirmed ICG-DNA-cho conjugation under Cy7 excitation (matching ICG emission spectrum). Nanoparticle tracking analysis (NTA) measurement showed that ICG@M2-exos had an average diameter of 130.255 ± 7.79 nm (Fig. 2E). TEM images confirmed the preserved spherical morphology with bilayer membrane structure (Fig. 2F). We employed cytotoxicity assay to validate the biosafety of these engineered exosomes for cellular applications. Both native M2-exos and ICG@M2-exos showed negligible cytotoxicity within a wide concentration range, confirming their excellent biocompatibility (Fig. 2G).

In summary, M2-exos and ICG@M2-exos were successfully fabricated with favorable immunomodulatory activity and low cytotoxicity, supporting their potential as biocompatible exosome-based platforms. Notably, hypoxia-induced M2-exos (1% O₂) exhibited significantly higher anti-inflammatory factor expression compared to normoxic controls, indicating superior inflammatory targeting and immunoregulatory capacity.

### Antibacterial properties of ICG@M2-exos

**Fig. 3.**
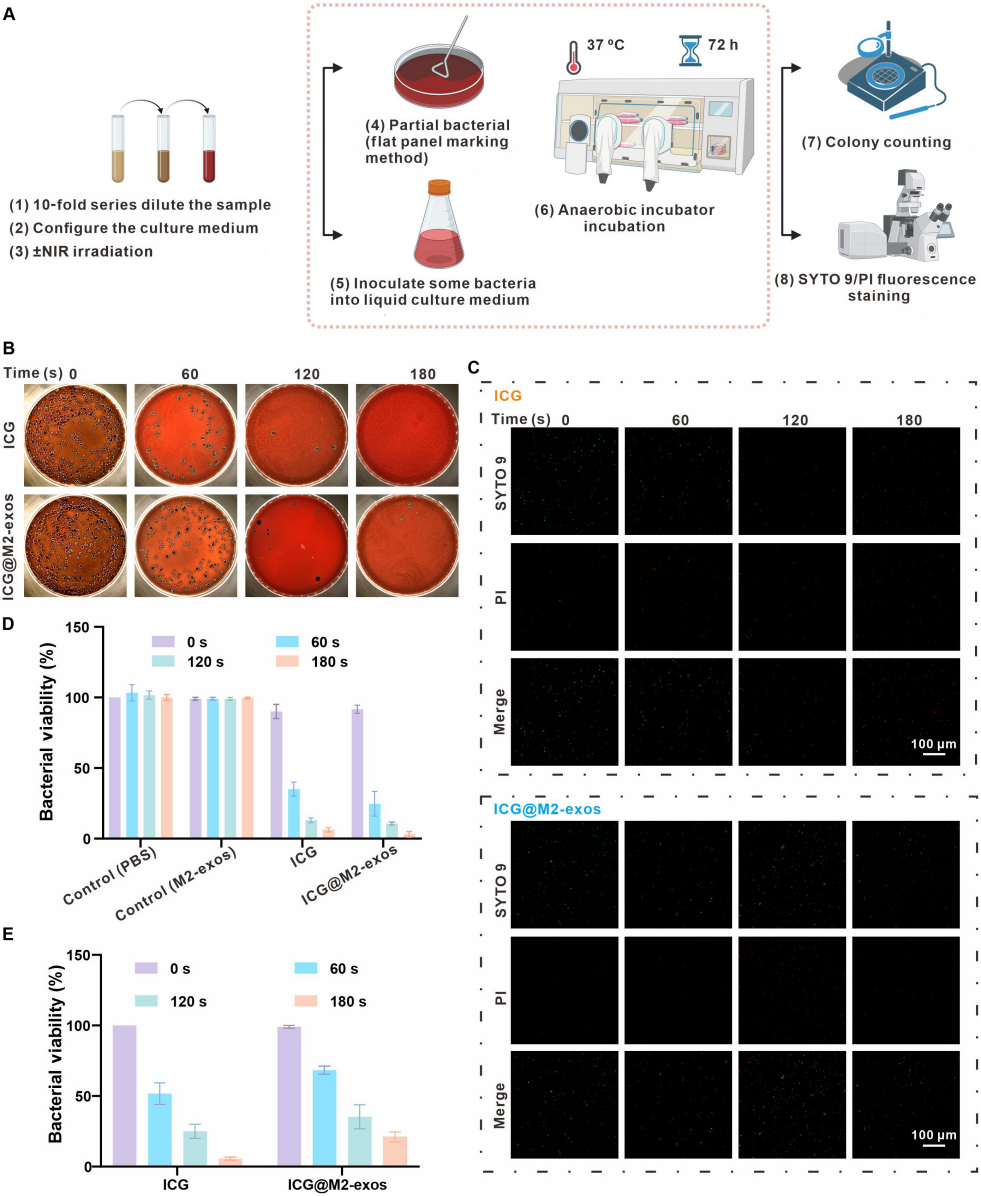
Assessment of the antibacterial efficacy of indocyanine green-loaded M2 macrophage-derived exosomes (ICG@M2-exos) against *Porphyromonas gingivalis* (*P. gingivalis*). (A) Schematic diagram illustrating the experimental timeline of the 3-d antibacterial assay. (B) The bacterial viability determined by plate spread assay. (C) Representative fluorescence micrographs of *P. gingivalis* after live/dead staining. Live bacteria with intact membranes stain green (SYTO 9), whereas bacteria with compromised membranes stain red (propidium iodide [PI]). Scale bar: 100 μm. (D) Quantitative analysis of bacterial viability determined by plate spread assay. The antibacterial efficacy was calculated relative to the ICG + near-infrared (NIR) group (defined as 100% efficacy). (E) Quantitative analysis of bacterial viability determined by live/dead staining. Data are presented as means ± SD (*n* = 3). PBS, phosphate-buffered saline.

The antimicrobial efficacy of ICG@M2-exos was assessed using a plate spread assay against *P. gingivalis*, a keystone pathogen in periodontitis [32]. A 3-d antibacterial assay was performed as schematically represented in Fig. 3A. Robust proliferation of *P. gingivalis* was observed in the untreated group, simulating the initial phase of periodontal infection. In contrast, treatment with ICG plus NIR irradiation completely eradicated all bacterial colonies, demonstrating potent antibacterial activity. This group was therefore designated as the positive control and assigned 100% antibacterial efficacy for reference (Fig. 3B; see Fig. 3D and Table S3 for statistical analysis; the important control groups with phosphate-buffered saline [PBS] and with M2-exos are shown in Fig. S1 and Table S2). The antibacterial efficacy of ICG@M2-exos + NIR was highly dependent on irradiation time. It achieved approximately 80% bactericidal efficiency relative to the positive control after 120-s irradiation. While extending the duration of irradiation to 180 s enhanced its effect to a level comparable to the ICG + NIR group, reducing the time to 60 s markedly diminished its efficacy, resulting in a 30% reduction regarding the antibactericidal efficiency. This outcome confirms that irradiation time is a significant factor that determines the antibacterial performance of ICG@M2-exos + NIR.

Having established the time-dependent antibacterial effect of ICG@M2-exos + NIR through quantitative colony counting assays, we next sought to visualize the bacterial viability directly. The live/dead staining results were entirely consistent with the quantitative data (Fig. 3C and the statistical analysis in Fig. 3E). The intermediate killing efficiency of approximately 80% of the bacteria for the ICG@M2-exos + NIR (120 s) group was mirrored in its fluorescence profile, which showed a level of red fluorescence (dead bacteria) that was significantly stronger than the untreated control but weaker than the ICG + NIR-positive control. In contrast to the control group, which displayed predominantly green fluorescence indicative of viable bacteria, the ICG@M2-exos + NIR group demonstrated a clear reduction in green signal and a concurrent rise in red fluorescence (marking dead bacteria). This intermediate signal profile, positioned between the control and the fully effective ICG + NIR groups, visually corroborates its partial bactericidal efficacy. The antibacterial efficacy of ICG@M2-exos is driven by the photodynamic generation of ROS from ICG upon NIR exposure. The resulting ROS outburst triggers extensive oxidative assault on vital bacterial structures, including the cell wall, lipids, and proteins. This irreversible damage rapidly compromises the structural integrity and essential functions, leading to effective bacterial eradication.

### In vitro ROS scavenging of ICG@M2-exos

**Fig. 4.**
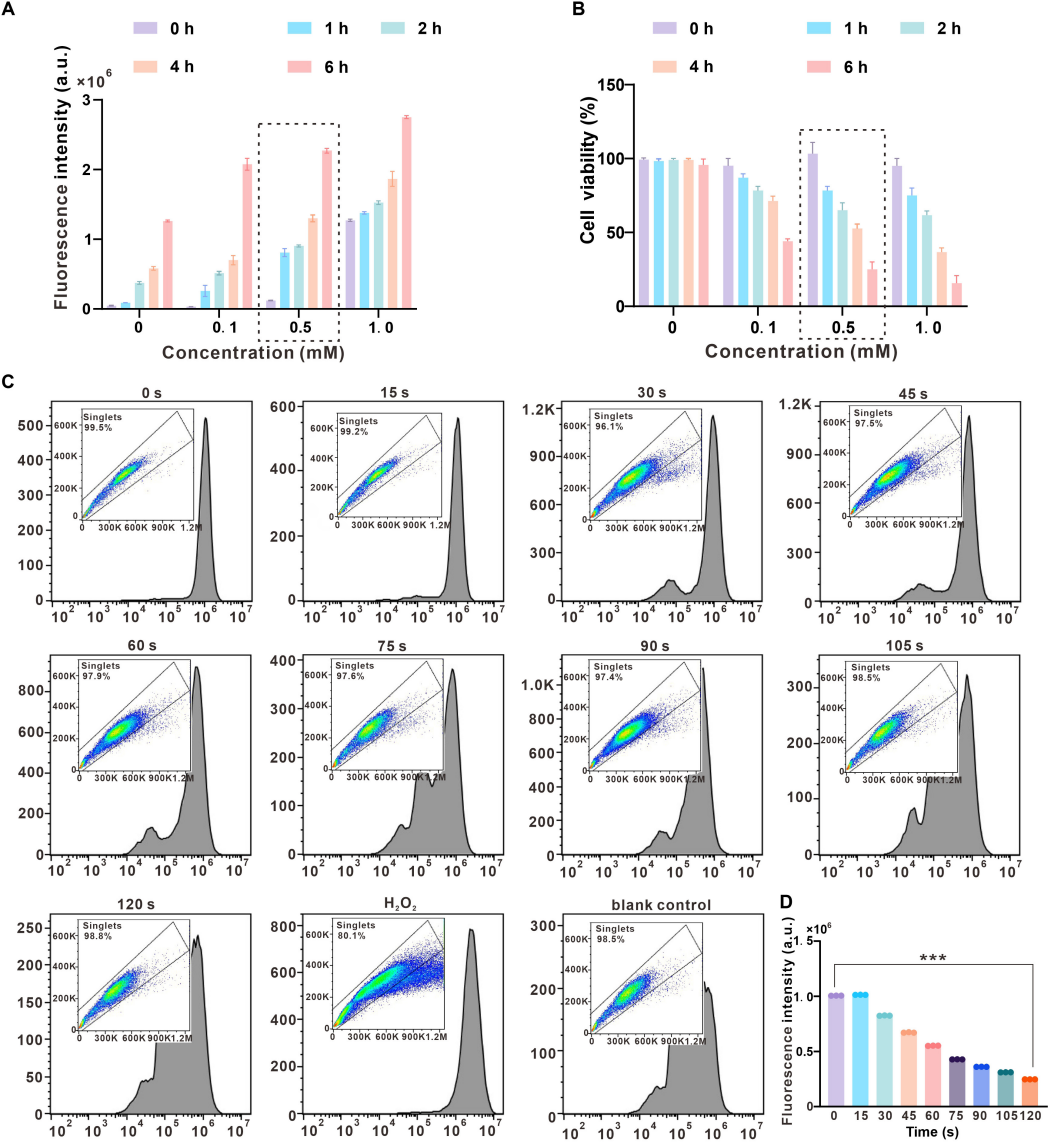
Indocyanine green-loaded M2 macrophage-derived exosomes (ICG@M2-exos) plus near-infrared (NIR) irradiation exhibits a time-dependent dual role in reactive oxygen species (ROS) regulation. (A) A dose- and time-dependent increase in intracellular ROS levels was detected upon H_2_O_2_ treatment (0.1 to 1.0 mM, 1 to 6 h). The 0.5 mM, 4-h condition (indicated) was chosen for subsequent studies. (B) Corresponding cell viability assessment for the selected condition, verifying a suitable window for intervention studies. (C) Fluorescence images showing intracellular ROS levels (green) under different conditions. (D) Quantitative fluorescence intensity analysis. Values are means ± SD (*n* = 3). ***P < 0.001. a.u., arbitrary units.

Excessive ROS accumulation is a key contributor to inflammatory pathogenesis, making efficient ROS scavenging a promising therapeutic strategy for various inflammatory diseases [33]. To establish a reliable in vitro oxidative stress model, we initially cultured HGF-1 cells in serum-free medium for 24 h. The main purpose was to reduce the basal metabolic level and endogenous antioxidant capacity of the cells, thereby making them more sensitive to subsequent oxidative stress stimuli. HGF-1 cells were then exposed to varying concentrations of H_2_O_2_ (ranging from 0.1 to 1.0 mM) for different time periods (1 to 6 h). The optimal induction condition was determined by measuring intracellular ROS levels via a fluorometric assay using a microplate reader. As summarized in Fig. 4A, both the concentration of H_2_O_2_ and the stimulation time significantly influenced ROS generation. A condition of 0.5 mM H_2_O_2_ for 4 h was selected for subsequent experiments, as it induced a substantial and consistent increase in fluorescence intensity, indicating robust oxidative stress without causing immediate overt cytotoxicity (cell viability staining ratio; Fig. 4B), thereby providing a suitable window for evaluating therapeutic interventions. Based on this optimized model, the intracellular ROS-scavenging capacity of ICG@M2-exos was then evaluated. Macrophages pretreated with 0.5 mM H_2_O_2_ for 4 h were incubated with ICG@M2-exos and subjected to NIR irradiation (808 nm, 500 mW/cm^2^) for varying durations (15 to 120 s). Intracellular ROS levels were assessed using the dichlorodihydrofluorescein diacetate (DCFH-DA) probe, which emits green fluorescence upon oxidation by ROS. As illustrated in Fig. 4C, macrophages in the H₂O₂-treated group exhibited intense green fluorescence, confirming successful induction of oxidative stress. In contrast, only minimal fluorescence was detected in the untreated control group without H_2_O_2_ stimulation. Notably, when H_2_O_2_-stimulated macrophages were subjected to ICG@M2-exos plus NIR irradiation (808 nm; 50 mW/cm²; 15 to 120 s), a marked reduction in ROS-associated fluorescence was observed. However, under shorter irradiation time (when exposed to NIR for 15 s), a slight increase in fluorescence intensity was detected, which might be attributed to the photodynamic effect of ICG generating additional ROS in the remaining viable cells. Quantitative analysis further supported these observations. The ICG@M2-exos + NIR treatment under 120-s irradiation demonstrated the highest ROS-scavenging efficacy, reducing the mean fluorescence intensity from 1.01 × 10^7^ in the H_2_O_2_-treated control group to 1.89 × 10^6^ (Fig. 4D). This result underscores the potent and time-dependent antioxidative capability of ICG@M2-exos under optimized phototherapeutic conditions.

### In vivo ROS scavenging of ICG@M2-exos

**Fig. 5.**
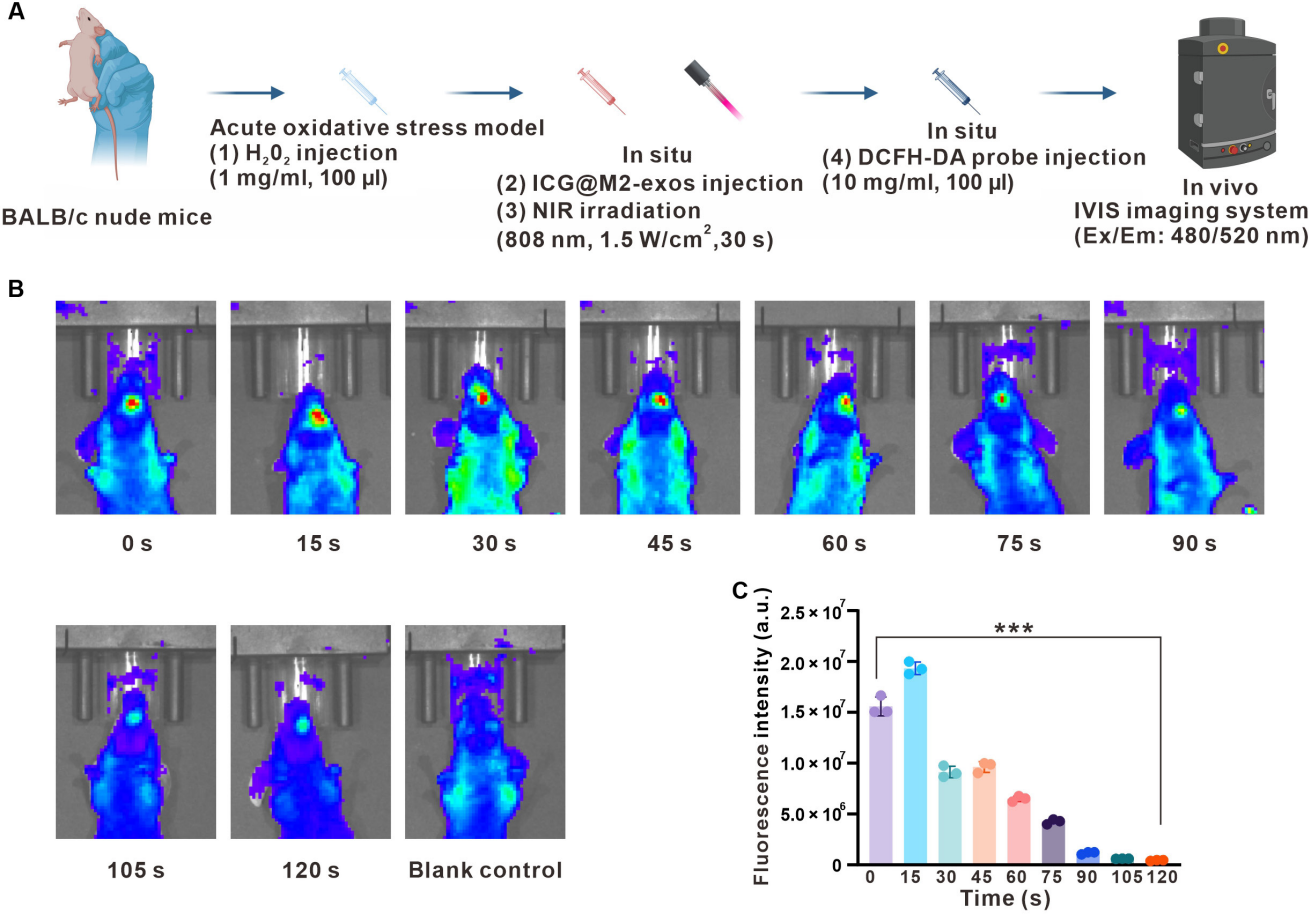
Validation of the therapeutic potential of indocyanine green-loaded M2 macrophage-derived exosomes (ICG@M2-exos) in mitigating oxidative stress in vivo. (A) Experimental scheme for establishing a localized H_2_O_2_-induced oxidative stress model in mouse gingival tissue and the subsequent therapeutic intervention with ICG@M2-exos and near-infrared (NIR). (B) Representative images of ICG@M2-exos–treated mice at different time points in comparison with the H_2_O_2_-treated mice. (C) Quantitative analysis. Data are means ± SD; ***P < 0.001. DCFH-DA, dichlorodihydrofluorescein diacetate; a.u., arbitrary units.

Building upon the established in vitro oxidative stress model, we further investigated the ROS-scavenging efficacy of ICG@M2-exos under pathophysiologically relevant conditions in vivo. To this end, a localized oxidative stress model [34] was generated by subcutaneous injection of H_2_O_2_ into the gingival tissue adjacent to the mandibular incisors of BALB/c nude mice (Fig. 5A). This intervention successfully induced a robust oxidative microenvironment, as visually evidenced by intense green fluorescence from the DCFH-DA probe at the injection site in the H_2_O_2_-treated group (Fig. 5C). Remarkably, subsequent local administration of ICG@M2-exos, followed by a precisely controlled NIR irradiation (808 nm, 1.5 W/cm^2^, 30 s), resulted in a dramatic attenuation of the fluorescent signal. Quantitative analysis corroborated this visual observation, revealing that the ICG@M2-exos + NIR treatment significantly reduced the mean fluorescence intensity by approximately 85% (Fig. 5B). This striking clearance of ROS in vivo not only validates the potent antioxidative capacity of ICG@M2-exos within a complex living system but also strongly mirrors the high efficiency observed in our prior in vitro assays, thereby confirming its therapeutic potential for mitigating oxidative stress-associated inflammatory damage.

The establishment of both in vitro and in vivo oxidative stress models is critically grounded in the well-documented pathogenesis of periodontitis, where excessive ROS accumulation acts as a key driver of inflammatory damage and tissue destruction. Our in vitro model confirmed the potent ROS-scavenging capability of ICG@M2-exos at the cellular level. Most importantly, the subsequent in vivo model, which locally induced oxidative stress in the gingival tissue, directly demonstrated that ICG@M2-exos coupled with NIR irradiation could effectively reduce ROS level within the complex tissue microenvironment. This successful reversal of oxidative stress in vivo not only validates the therapeutic potential of ICG@M2-exos but also provides a compelling rationale for the next logical step of our investigation.

### In vitro immunomodulation of ICG@M2-exos

Given the close link between oxidative stress and the propagation of inflammation [35], we next sought to determine whether the potent antioxidative effect of ICG@M2-exos could consequently translate into meaningful anti-inflammatory outcomes. We therefore systematically evaluated its immunomodulatory performance in both an in vitro macrophage model and the established in vivo periodontitis model. An in vitro inflammation model was established by stimulating THP-1 cells with *P. gingivalis*-derived lipopolysaccharide (LPS) to induce M1 macrophage polarization [36]. The LPS-treated group, designated Control (+), served as the 100% reference baseline. To evaluate the immunomodulatory effect of different treatments, mRNA expression of key cytokines was quantified via quantitative polymerase chain reaction, followed by measurement of corresponding protein secretion using enzyme-linked immunosorbent assay (ELISA). The inflammatory response involves dynamic shifts in macrophage polarization. Early-stage proinflammatory M1 macrophages secrete cytokines such as tumor necrosis factor-α (TNF-α) and interleukin-1β (IL-1β), driving catabolic tissue destruction. While critical for host defense, sustained M1 activation leads to chronic tissue damage. Thus, the M1-to-M2 phenotypic transition is essential for inflammation resolution and tissue repair. M2 macrophages secrete interleukin-10 (IL-10) and arginase-1 (Arg-1), promoting anabolic reconstruction and healing, highlighting the exquisite balance between M1/M2 response in determining pathological outcomes.

**Fig. 6.**
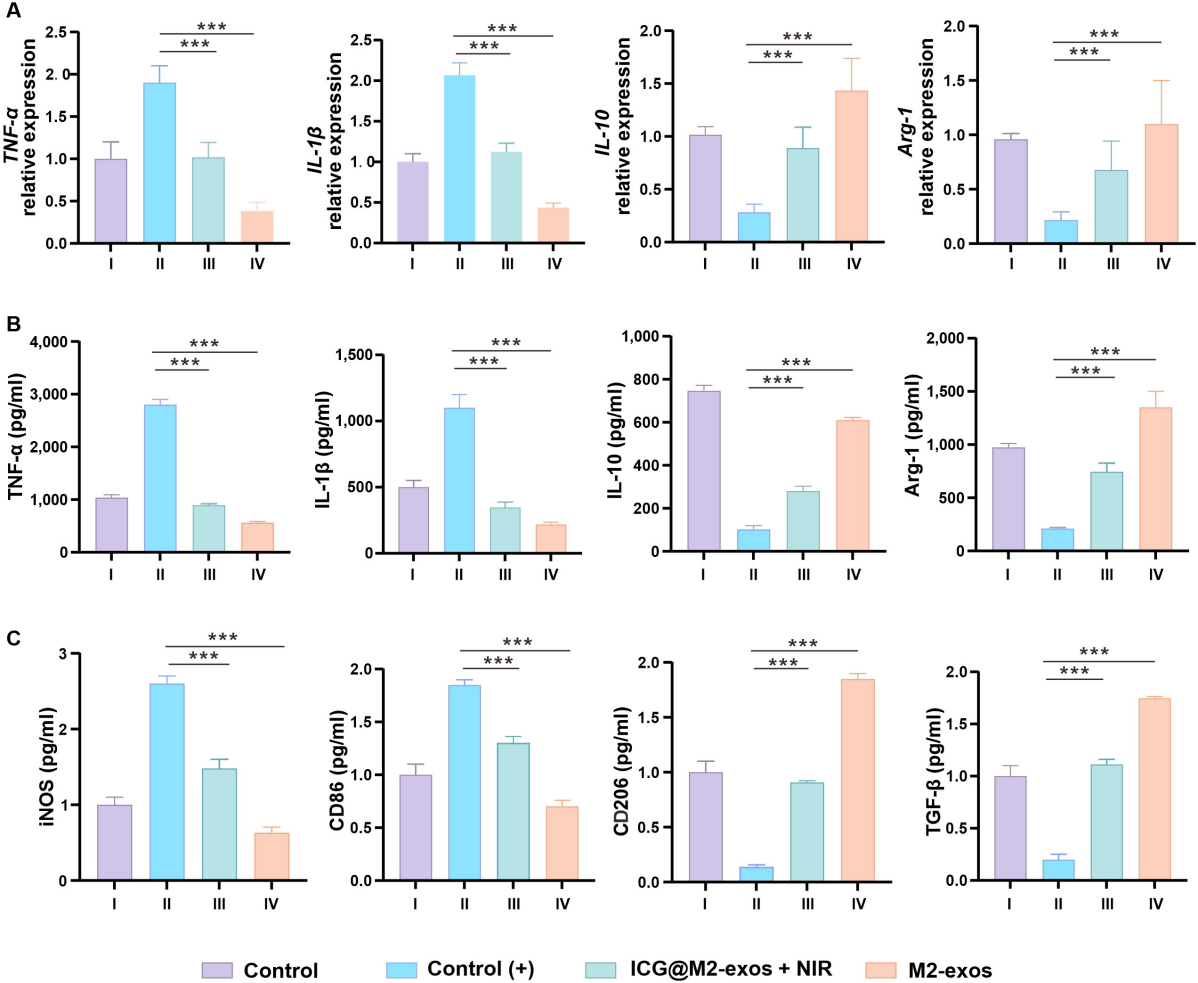
Indocyanine green-loaded M2 macrophage-derived exosomes (ICG@M2-exos) combined with near-infrared (NIR) irradiation reprograms macrophage polarization by synchronously modulating gene and protein expression of key cytokines. (A) mRNA (M1: *TNF-α* and *IL-1β*; M2: *IL-10* and *Arg-1*) and (B) protein (M1: tumor necrosis factor-α [TNF-α] and interleukin-1β [IL-1β]; M2: interleukin-10 [IL-10] and arginase-1 [Arg-1]) expression levels of macrophage polarization markers in *Porphyromonas gingivalis* (*P. gingivalis*)-lipopolysaccharide (LPS)-stimulated THP-1 macrophages. (C) Temporal profiling of polarization markers confirmed a shift from M1 (inducible nitric oxide synthase [iNOS] and cluster of differentiation 86 [CD86]) to M2 (cluster of differentiation 206 [CD206] and transforming growth factor-β [TGF-β]) phenotypes, underscoring the mechanism of action. Data are means ± SD (*n* ≥ 3). ***P < 0.001 versus the LPS group.

As anticipated, stimulation with *P. gingivalis*-derived LPS significantly up-regulated *TNF-α* and *IL-1β* at the mRNA levels and TNF-α and IL-1β at the protein levels in THP-1 cells after 24 h (Fig. 6A and B). This proinflammatory response was markedly suppressed by ICG@M2-exos after coculturing with THP-1 cells for 48 h, achieving nearly 80% reduction. The ICG@M2-exos + NIR treatment retained 70% to 80% of M2-exos’s inhibitory potency, confirming preserved efficacy of the combined modality. Conversely, all treatments significantly enhanced anti-inflammatory mediators IL-10 and Arg-1 expression: M2-exos alone induced ~5-fold up-regulation, while ICG@M2-exos + NIR maintained ~60% to 65% of this enhancement. Protein levels closely mirrored transcriptional changes, affirming consistent regulation across molecular tiers.

To further validate the M1-to-M2 phenotypic shift, we analyzed a comprehensive set of polarization markers after 48 h of cell coculture. M1 markers (inducible nitric oxide synthase and cluster of differentiation 86 [CD86]) were progressively down-regulated, while M2 markers (cluster of differentiation 206 [CD206] and transforming growth factor-β) were concurrently up-regulated (Fig. 6C). A distinct efficacy gradient emerged: M2-exos induced the most robust transition, whereas ICG@M2-exos achieved 50% to 60% of M2-exos’s effect. Both treatments, however, significantly promoted a prorepair profile compared to Control (+), confirming their differential yet substantial role in phenotype reprogramming.

Collectively, these results demonstrate that ICG@M2-exos, especially when combined with NIR, synergistically reprograms macrophages from M1 to M2 phenotype. This immunomodulatory shift underpins a potent therapeutic strategy for mitigating chronic inflammation, positioning ICG@M2-exos as a promising candidate for periodontitis treatment.

### In vivo immunomodulation of ICG@M2-exos

**Fig. 7.**
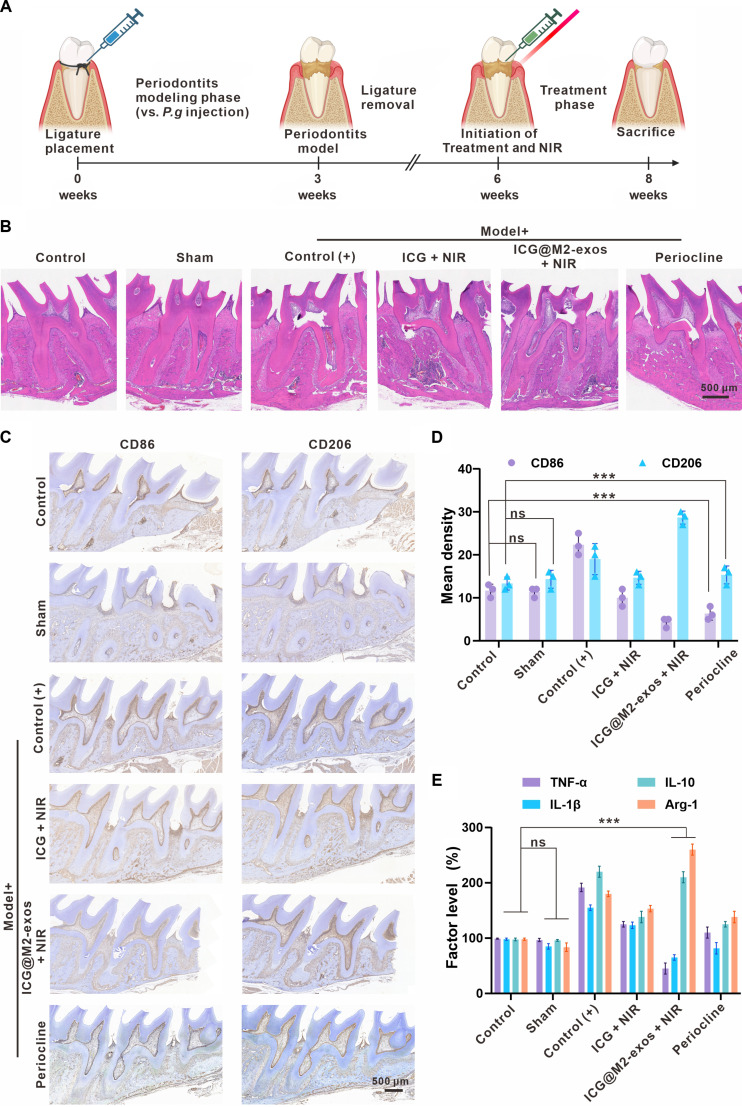
Indocyanine green-loaded M2 macrophage-derived exosomes (ICG@M2-exos) + near-infrared (NIR) treatment alleviates periodontitis and induces macrophage M2 polarization in rats. (A) Experimental timeline. (B) Hematoxylin and eosin (H&E) staining showed severe alveolar bone resorption and inflammatory infiltration in the Control (+) group, which were markedly attenuated in the ICG@M2-exos + near-infrared (NIR) group. Scale bar: 500 μm. (C) Immunohistochemistry for cluster of differentiation 86 (CD86) (M1) and cluster of differentiation 206 (CD206) (M2) revealed a predominant M1 phenotype in Control (+) group, while ICG@M2-exos + near-infrared (NIR) promoted a shift to an M2 phenotype. Scale bar: 500 μm. (D) ImageJ analysis to quantify the immunohistochemistry staining. ***P < 0.001. (E) Enzyme-linked immunosorbent assay (ELISA) analysis of gingival crevicular fluid indicated that ICG@M2-exos + NIR significantly reduced the expression of proinflammatory cytokines (tumor necrosis factor-α [TNF-α] and interleukin-1β [IL-1β]) and increased the expression of anti-inflammatory factors (interleukin-10 [IL-10] and arginase-1 [Arg-1]). ***P < 0.001. ns, not significant.

An inflammatory model of periodontitis in Sprague–Dawley rats was established through local injection of *P. gingivalis* bacterial fluid into the gingival sulcus [37]. Following an 8-week treatment phase, the rats were humanely sacrificed for further analyses. The overall experimental timeline is depicted in Fig. 7A, and a photographic record of the model assembly process is provided in Fig. S2. During the entire experimental period, a systematic in vivo biosafety evaluation was carried out. Histopathological examination of major organs (heart, liver, spleen, lungs, and kidneys) via hematoxylin and eosin (H&E) staining, in conjunction with hematological toxicity analysis, indicated no significant treatment-associated abnormalities in any of the groups, as presented in Fig. S3.

H&E staining of periodontal tissues revealed distinct pathological changes across groups (Fig. 7B), and higher-magnification H&E images (Fig. S4). The Control (+) group exhibited severe alveolar bone resorption, disrupted periodontal ligament, irregular bone crest, and dense inflammatory cell infiltration, consistent with advanced periodontitis. In contrast, the ICG@M2-exos + NIR group showed marked histological recovery, with significantly reduced inflammatory cell infiltration, preserved tissue architecture, and attenuated bone erosion. Immunohistochemistry analysis further demonstrated that this recovery resulted from the reprogramming of local immune microenvironment. Tissue from the Control (+) group was dominated by CD86^+^ M1 macrophages, with few CD206^+^ M2 cells detected (Fig. 7C). ICG@M2-exos + NIR treatment strikingly reversed this profile, almost eliminating all CD86^+^ cells while strongly enhancing CD206^+^ expression, indicating a shift from M1 to M2 polarization. The negligible effect of ICG + NIR alone underscored the essential role of M2-exos cargo in this immunomodulating process. Cytokine profiling of gingival crevicular fluid by ELISA corroborated the above findings (Fig. 7D). The Control (+) group showed high proinflammatory (TNF-α and IL-1β) and low anti-inflammatory (IL-10 and Arg-1) expression levels (Fig. 7E). ICG@M2-exos + NIR significantly reversed this profile, concurrently suppressing the expression of proinflammatory factors while enhancing anti-inflammatory ones. Although Periocline also reduced the expression level of proinflammatory cytokines, it did not comparably elevate IL-10 or Arg-1 expression, highlighting the dual-phase immunomodulatory capacity of ICG@M2-exos + NIR in restoring the local immune homeostasis.

Collectively, our in vivo results demonstrate that ICG@M2-exos + NIR mitigates periodontitis through a multipronged mechanism: reducing inflammatory infiltration while promoting collagen reorganization, via promoting a decisive M1-to-M2 macrophage transition. This rebalancing process of the periodontal microenvironment underscores the potential of ICG@M2-exos as a next-generation immunomodulatory therapy for treating chronic periodontitis.

### Inhibition of alveolar bone resorption by ICG-loaded M2-exosomes

**Fig. 8.**
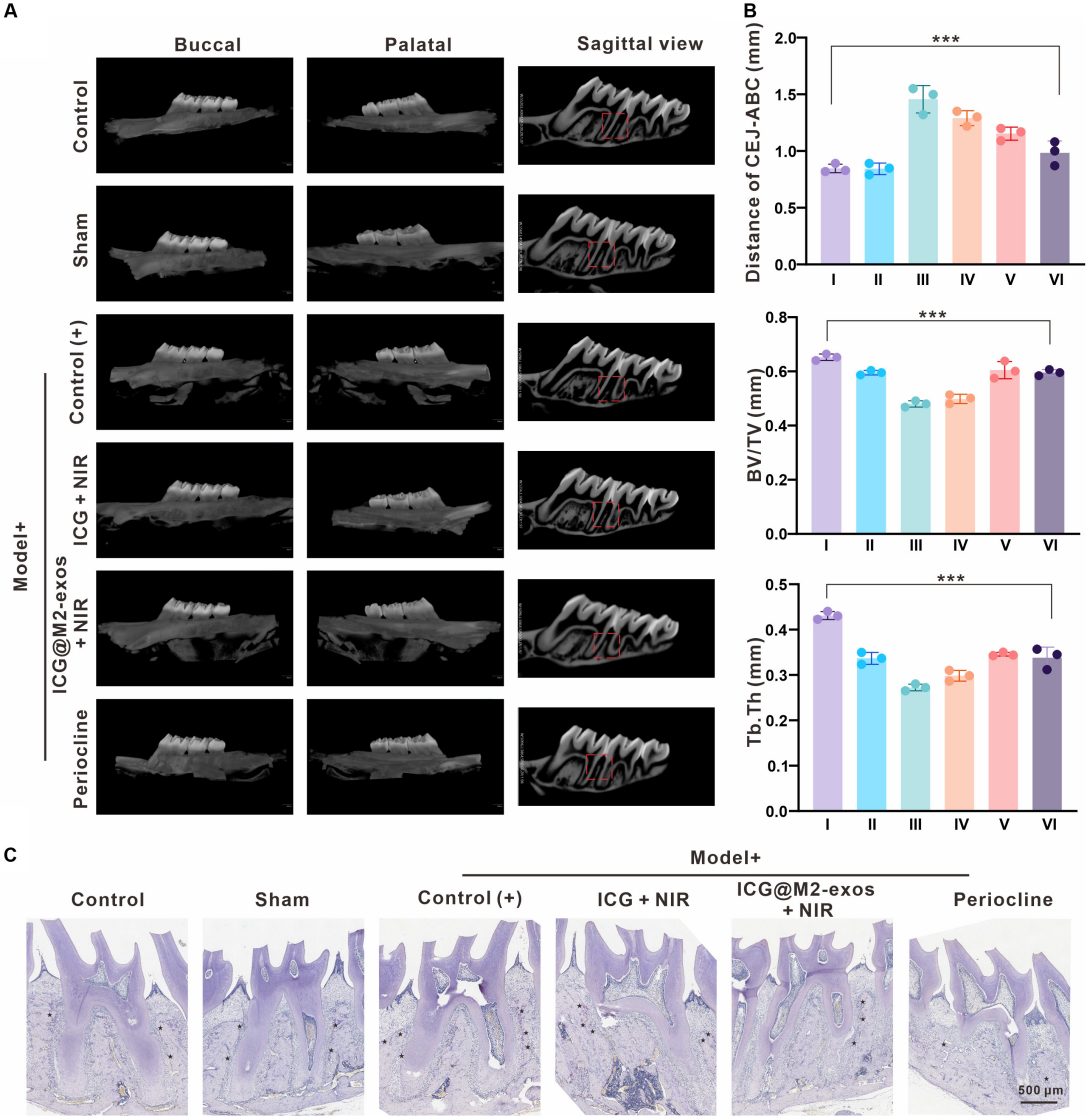
Evaluation of alveolar bone microstructure and osteoclast activity. (A) Representative 2-dimensional (2D) and 3-dimensional (3D) micro-computed tomography (micro-CT) reconstructions of the alveolar bone. (B) Quantitative microstructural parameters: cementoenamel junction-alveolar bone crest (CEJ-ABC) distance, bone volume/total volume (BV/TV), and trabecular thickness (Tb.Th). (I) Control; (II) sham; (III) Control (+); (IV) indocyanine green (ICG) + near-infrared (NIR); (V) ICG-loaded M2 macrophage-derived exosomes (ICG@M2-exos); (VI) Periocline). ***P < 0.001. (C) Tartrate-resistant acid phosphatase (TRAP) staining of alveolar bone sections to show bone resorption pits (black asterisks) and osteoclasts (red staining). Scale bar: 500 μm.

The therapeutic efficacy of indocyanine green-loaded M2 exosomes (ICG@M2-exos) was further validated by its robust protective effect against alveolar bone destruction. Micro-computed tomography (micro-CT) analysis (Fig. 8A and B) confirmed that ICG@M2-exos combined with NIR irradiation significantly preserved bone microstructure, characterized by increased bone volume fraction (bone volume/total volume) (BV/TV) and reduced cementoenamel junction-alveolar bone crest (CEJ-ABC) distance. Concomitantly, tartrate-resistant acid phosphatase (TRAP) staining (Fig. 8C) demonstrated a marked reduction in osteoclast number and resorptive activity in the ICG@M2-exos + NIR group, directly reflecting the restoration of bone surface integrity. These findings indicate that the immunomodulatory strategy of ICG@M2-exos combined with NIR irradiation can inhibit osteoclast activation, thereby achieving structural and functional preservation of the alveolar bone.

Alveolar bone morphology was then evaluated via 2-dimensional/3-dimensional micro-CT reconstruction (Fig. 8A). Quantitative parameters including CEJ-ABC distance, bone volume fraction (BV/TV), and trabecular thickness (Tb.Th) were all measured to assess alveolar bone loss (Fig. 8B). The untreated periodontitis group [Control (+)] exhibited severe alveolar bone resorption, particularly at the buccal/palatal aspects of the second molar (M2). Micro-CT analysis confirmed the most severe bone loss in this group (CEJ-ABC: 1.55 ± 0.18 mm; Tb.Th: 0.272 ± 0.017 mm; BV/TV: 0.480 ± 0.011), validating successful periodontitis model establishment. In contrast, the ICG@M2-exos + NIR group showed the most significant alveolar bone restoration. After 8-week intervention, CEJ-ABC distance was reduced to 78% ± 2% when compared to the Control (+) level, indicating mitigated bone resorption. Tb.Th and BV/TV increased to 122.98% ± 0.02% and 122.45% ± 0.06% compared to the Control (+) value, respectively, reflecting improved trabecular structure and bone density.

Periodontitis-associated alveolar bone loss arises from uncoupled bone remodeling, pathologically exacerbated by excessive osteoclast activity. Inhibiting osteoclastogenesis thus represents a rational therapeutic strategy to mitigate inflammatory bone destruction. TRAP staining (Fig. 8C) confirmed this pathophysiological mechanism: the Control (+) group exhibited dense TRAP-positive multinucleated cells (red-stained, star-indicated) with associated resorption pits on the alveolar bone surface. In sharp contrast, the ICG@M2-exos + NIR group showed nearly complete osteoclast elimination, consistent with previous micro-CT observations of preserved bone microarchitecture. These findings indicate that ICG@M2-exos + NIR alleviates periodontitis through dual mechanisms: inflammation modulation and direct suppression of osteoclast-mediated bone resorption, highlighting its therapeutic potential.

## Conclusion

In this study, we engineered a bioinspired nanoplatform based on ICG-loaded M2 macrophage-derived exosomes (ICG@M2-exos) for synergistic periodontitis therapy. This nanosystem integrates 3 core functionalities: NIR-responsive photodynamic bactericidal activity via ICG-mediated ROS generation, immunomodulation through M2-exosomal cargo, and oxidative stress mitigation by exosome-derived antioxidant enzymes. In vitro experiments confirmed potent bactericidal efficacy against *P. gingivalis* and precise regulation of macrophage polarization. In a rat periodontitis model, local administration of ICG@M2-exos significantly reduced gingival inflammation, suppressed proinflammatory cytokine expression, and preserved alveolar bone structure by inhibiting osteoclast activity. The exosome-based platform offers inherent biocompatibility, low immunogenicity, and deep tissue penetration, addressing limitations of conventional monotherapies. ICG@M2-exos achieve this through initial photodynamic bactericidal activity under NIR irradiation, followed by exosome-mediated immunomodulation and oxidative stress relief, which collectively suppress osteoclastogenesis and promote alveolar bone regeneration. Collectively, they exert multimodal therapeutic effects by targeting key pathological hallmarks of periodontitis, presenting a promising nanotherapeutic strategy for periodontitis and other inflammatory bone disorders with similar pathogen-immune-osteoclast interactions.

## Materials and Methods

### Isolation and characterization of M2-exos

The human monocytic cell line THP-1 (TCHu 57, Cell Bank of Typical Culture Preservation Committee of Chinese Academy of Sciences) was used in this study. Prior to polarization, the cells were differentiated into macrophages via a 48-h exposure to 100 ng/ml PMA (HY-18739, MCE, USA). Subsequent polarization into the M2 phenotype was accomplished by treating the differentiated macrophages for 24 h with a cytokine cocktail containing 20 ng/ml of both IL-4 (HY-P70445, MCE, USA) and IL-13 (HY-P70568, MCE, USA). To generate the experimental group, these M2 macrophages were then subjected to hypoxic preconditioning in a specialized hypoxic chamber (1% O₂, 5% CO₂, 94% N₂) for 24 h. The control group (normoxic M2 macrophages) was maintained under standard culture conditions (21% O₂, 5% CO₂). Exosomes were isolated from the cell culture supernatants of both hypoxic-preconditioned and normoxic M2 macrophages. M2-exosomes (M2-exos) were isolated from the conditioned medium using the ExoQuick ULTRA kit (EQULTRA-20A-1, SBI, USA) according to the manufacturer’s instructions. Briefly, the supernatant was centrifuged to remove cells and debris, mixed with ExoQuick solution, and incubated overnight at 4 °C. The mixture was then centrifuged to obtain the exosome pellet, which was subsequently resuspended in PBS. The isolated M2-exos underwent rigorous characterization to confirm their identity and quality. First, morphological examination was conducted via TEM (JEM-1400Flash, JEOL, Japan). Second, the hydrodynamic diameter and concentration were assessed by NTA (ZetaVIEW S/N 21-610, PMX, Germany). Third, Western blot analysis was performed to detect the expression of specific exosomal surface antigens, namely, CD63 and CD81. M2-exos preparations that satisfactorily met these quality control standards were advanced for use in downstream studies.

### Preparation of ICG@M2-exos

The photosensitizer ICG (HY-D0711, MCE, USA) was loaded onto the surface of M2-exos via a cholesterol (cho)-mediated anchor as illustrated in Fig. 1A, with the preparation performed in a 2-step procedure: First, the ICG-DNA-cho conjugate was prepared through an amide coupling reaction between NH₂-DNA-cho and ICG-NHS at a molar ratio of 1:1.5 in 0.1 M sodium bicarbonate buffer (pH 8.5) for 6 h at room temperature in the dark, and the product was purified using a 3-kDa centrifugal filter to remove unreacted reagents, yielding a dark green solution; second, the ICG-DNA-cho conjugate was incubated with M2-exos to allow anchoring of ICG onto the exosomal surface via hydrophobic interactions between the cholesterol moiety and the exosomal membrane. After incubation at 37 °C for 1 h, unbound conjugates were removed by washing with PBS and ultracentrifugation (100,000 × g, 70 min, 4 °C). The resulting ICG@M2-exos pellet was resuspended in PBS and stored at 4 °C in the dark, and successful construction was confirmed by NTA for size and concentration, TEM for morphology, and immunofluorescence showing colocalization of ICG with PKH26-labeled M2-exos.

### Cell culture

The present study employed the human monocytic THP-1 cell line. The cells were cultured in RPMI 1640 medium (Gibco, USA) supplemented with 30 ng/ml macrophage colony-stimulating factor (PeproTech, USA) and maintained at 37 °C in a 5% CO₂ humidified incubator. For routine subculture, suspended THP-1 cells were harvested by centrifugation at 300 × g for 5 min and reseeded in fresh complete medium at densities ranging from 2 × 10^5^ to 1 × 10^6^ cells/ml to ensure continuous logarithmic growth. To initiate differentiation into macrophage-like cells, THP-1 cells were plated and activated with 100 ng/ml PMA for 48 h. Following this treatment, the PMA-containing medium was aspirated, and the resulting adherent macrophage monolayer was gently rinsed twice with PBS to remove nonadherent cells and residual reagent. Prior to subsequent experiments, the differentiated macrophages were then maintained in PMA-free complete medium for an additional 24 h to attain a quiescent state.

### Plate counting assay (CFU assay)

*P. gingivalis* (American Type Culture Collection 33277) was anaerobically cultured in brain heart infusion broth (B8130, Solarbio, China) supplemented with hemin (5 mg/l; H6390, Sigma-Aldrich, USA), vitamin K1 (10 mg/ml; V3501, Sigma-Aldrich, USA), and 5% sterile defibrinated sheep blood (R54020, ThermoFisher, USA). Mid-logarithmic phase cultures were harvested by centrifugation, resuspended in fresh PBS, and adjusted to ~1 × 10^8^ colony-forming units (CFU)/ml for subsequent experiments. Bacterial suspensions were coincubated with different precontact culture media (ICG and ICG@M2-exos) and different precontact culture times (1, 3, 6, and 12 h) for a predetermined period. NIR laser irradiation was applied at 0, 60, 120, and 180 s to evaluate time-dependent photodynamic effects. After incubation, suspensions were serially diluted 10-fold in sterile PBS. Aliquots (100 μl) of appropriate dilutions were spread on prereduced brain heart infusion agar plates and anaerobically incubated at 37 °C for 3 d. Colonies were counted, and results were expressed as CFU/ml {Antibacterial Rate (%) = [(CFU control − CFU treatment) / CFU control] × 100%}.

### Bacterial live/dead staining assay

Viability of *P. gingivalis* was assessed using a Live/Dead BacLight Bacterial Viability Kit for qualitative and quantitative analyses. In accordance with the manufacturer’s instructions, bacteria were stained with a solution containing SYTO 9 and propidium iodide. SYTO 9 labels all cells green, while propidium iodide labels membrane-damaged cells red and quenches SYTO 9 fluorescence. After a 20-min dark incubation, samples were mounted on slides and immediately imaged using a confocal laser scanning microscope (A1R, Nikon, Japan). The resultant live (green) and dead (red) fluorescence signals were quantified via image analysis to determine the live-to-dead ratio.

### In vitro H_2_O_2_-induced oxidative stress model

HGF-1 cells (American Type Culture Collection CRL-2014) were seeded and pretreated with test formulations (serum-free medium preincubation, 24 h). Oxidative stress was induced by replacing culture medium with 500 μM H₂O₂ (preliminary optimized to achieve ~50% cell mortality), followed by 4 h of incubation. Intracellular ROS detection: Intracellular ROS levels were quantified using DCFH-DA (HY-D0940, MCE, USA). Post H₂O₂ challenge, cells were incubated with DCFH-DA (37 °C, 30 min, dark). DCF fluorescence was detected at 488 nm via fluorescence-activated cell sorting flow cytometry (BD Biosciences, USA), and fluorescence intensity was quantified using ImageJ (Media Cybernetics, USA).

### In vivo ROS detection via live animal imaging

A localized oxidative stress model was constructed in the periodontal tissue of BALB/c nude mice through the daily injection of H₂O₂ solution (1 mg/ml, 100 μl) into the gingival sulcus of the mandibular incisors for 3 consecutive days. Thirty minutes prior to treatment, the DCFH-DA probe (10 mg/ml, 100 μl) was injected in situ at the identical site. After a 30-min probe incubation period, the respective therapeutic formulations were administered in accordance with the experimental groups, followed by NIR laser irradiation at various durations (0, 15, 30, 45, 60, 75, 90, 105, and 120 s). Subsequently, fluorescence images of the jaw region were captured using an IVIS imaging system (excitation/emission: 480/520 nm). The mean fluorescence intensity within the region of interest surrounding the mandibular incisors was quantified using the instrument’s software, facilitating a direct comparison of ROS levels among different treatment groups.

### In vitro anti-inflammatory assessment on THP-1 macrophages

Cell culture and polarization: Macrophages were generated from THP-1 monocytes via PMA-driven differentiation (100 ng/ml, 48 h). These macrophages were then induced to adopt an M1 phenotype through a 24-h exposure to 100 ng/ml *P. gingivalis* LPS (*P.g*-LPS). Treatment groups: The M1-polarized macrophages (M1 macrophages collected from THP-1 cells cocultured with *P.g*-LPS for 24 h without any other treatment) were divided into the following treatment groups: (a) Control (THP-1 cells collected after PMA treatment, without subsequent stimulation with *P.g*-LPS): incubated with PBS; (b) Control (+): incubated with PBS; (c) M2-exos group: treated with M2-exos; (d) ICG@M2-exos group: treated with ICG@M2-exos (with or without NIR laser irradiation as per experimental design). Quantitative real-time polymerase chain reaction analysis: Following treatments, total RNA was extracted with TRIzol and reverse-transcribed into cDNA. Quantitative real-time polymerase chain reaction was performed to assess the expression of M1 (TNF-α and IL-1β) and M2 (IL-10 and Arg-1) macrophage phenotype markers, with GAPDH as the reference gene (Table S1). The 2^–ΔΔCt method was applied to determine relative expression. Protein secretion analysis by ELISA: Cell culture supernatants were harvested by centrifugation. The secretion levels of TNF-α, IL-1β, IL-10, Arg-1, inducible nitric oxide synthase, and transforming growth factor-β and expression level of surface markers (CD86 and CD206) were quantified using specific commercial ELISA kits, in strict adherence to the manufacturers’ protocols. The absorbance was measured at 450 nm, and cytokine concentrations were interpolated from their respective standard curves.

### In vivo anti-inflammatory assessment in a *P. gingivalis*-induced periodontitis rat model

Animal model establishment: Periodontitis was induced in Sprague–Dawley rats over 3 weeks: maxillary second molar gingival sulci were ligated with silk sutures for 2 h to facilitate bacterial invasion. The silk thread was removed after 2 h, followed by antibiotic suppression of native flora. “*P. gingivalis*” suspension (10^9^ CFU in 2% carboxymethylcellulose) was topically applied to prepared sulci multiple times weekly for 3 weeks to establish chronic inflammation. To ensure consistency and comparability, *P. gingivalis* cultures used for both antibacterial assays and animal model induction were prepared under identical conditions. Experimental groups and treatment: Periodontitis-induced rats were randomized into 5 groups: (a) Sham (no induction + PBS); (b) Control (+) (model + PBS); (c) ICG + NIR; (d) ICG@M2-exos + NIR; (e) Periocline. Formulations were administered via intragingival injection around target molars. Specifically, 200 μl of ICG@M2-exos suspension was applied topically to the prepared gingival sulcus per tooth per administration, 3 times weekly for 3 weeks.

At treatment end point, gingival crevicular fluid was collected from target molar sulci using sterile filter paper strips. Eluates were analyzed for TNF-α, IL-1β, IL-10, and Arg-1 concentrations via rat-specific ELISA kits. After 8 weeks of treatment, maxillae from euthanized rats were processed for IHC: Consecutive sections were stained for M1 marker CD86 and M2 marker CD206 using specific primary antibodies, followed by horseradish peroxidase-conjugated secondary antibodies and 3,3’-diaminobenzidine visualization with hematoxylin counterstaining. CD86^+^and CD206^+^cells in periodontal ligament were counted in 5 random high-power fields per sample, and M1/M2 ratio was calculated as CD86^+^/CD206^+^ cell count quotient (*n* = 6 per group). Major organs (heart, liver, spleen, lungs, and kidneys) were harvested for H&E staining to assess systemic toxicity. Maxillae were analyzed for periodontal tissue damage/repair via H&E (inflammatory infiltration and tissue structure), TRAP (osteoclast activity and bone resorption), and Masson staining (collagen deposition and fibrous regeneration around maxillary second molars).

### Statistical analysis

Data from a minimum of 3 independent replicates are presented as means ± SD. Intergroup differences across multiple conditions were assessed by 1-way analysis of variance (ANOVA) coupled with Tukey’s test for post hoc comparisons, implemented in GraphPad Prism (v9.0). Differences with a *P* value below 0.05 were deemed statistically significant. The specific statistical tests applied are detailed in the respective figure legends.

## Ethical Approval

All animal experiments were performed under protocols approved by the Institutional Animal Care and Use Committee of the Nanjing Medical University (IACUC-2310022). Moreover, approval was received prior to beginning this research.

## Data Availability

All data generated or analyzed during this study are included in this article and its supplementary files.
