# Supplementary material for: NIR-Activated ICG-Loaded M2 Macrophage Exosomes Ameliorate Periodontitis via Targeting Infection Inflammation and Oxidative Stress
Source: Research (Wash D C). 2026 Apr 27;9:1207. doi: 10.34133/research.1207 (PMC13113311; doi:10.34133/research.1207)
Supplement: Supplementary 1 — Figs. S1 to S4 Tables S1 to S4 [file research.1207.f1.zip › Supporting Information.docx]

**Supporting Information**

**An Integrated Strategy for Periodontitis: Engineered M2 Exosomes Loaded with ICG Simultaneously Tackling Infection, Inflammation, and Oxidative Stress**

Xincong Li^1†^, Xin Fu^1†^, Tianyu Zhang^1^, Xiaofan Cheng^1^, Hai Zhuang^1^, Zhilei Mao^2^*, Shoushan Bu^1^*

^1^Department of Stomatology, the First Affiliated Hospital of Nanjing Medical University, Nanjing 210029, P.R. China.

^2^Changzhou Maternity and Child Health Care Hospital, Changzhou Medical Center, Nanjing Medical University, Changzhou 213003, Jiangsu, P.R. China.

*Address correspondence to: [mao598808386@126.com](mailto:mao598808386@126.com) (Z.M.); [bushsh@vip.sina.com](mailto:bushsh@vip.sina.com) (S.B.).

^†^These authors contributed equally to this work.

**Table S1**

| **Gene Name** | **Forward Primer (5'→3')** | **Reverse Primer (5'→3')** | **Product Size (bp)** |
| --- | --- | --- | --- |
| ****TNF-α**** | ACTCCAGGCGGTGCCTATGT | GTGAGGGTCTGGGCCATAGAA | 160 |
| ****IL-1β**** | TCCAGGATGAGGACATGAGCAC | GAACGTCACACACCAGCAGGTTA | 105 |
| ****IL-10**** | TCAGGCTGAGGCTACGG | AGATGTCAAACTCACTCATGGC | 75 |
| ****Arg-1**** | ACATTGGCTTGCGAGACGTA | ATCACCTTGCCAATCCCCAG | 109 |
| ****GAPDH**** | TGTGTCCGTCGTGGATCTGA | TTGCTGTTGAAGTCGCAGGAG | 150 |

The primer sequences used for the amplification of TNF-α, IL-1β, IL-10, Arg-1, and the reference gene GAPDH in THP-1 cells.


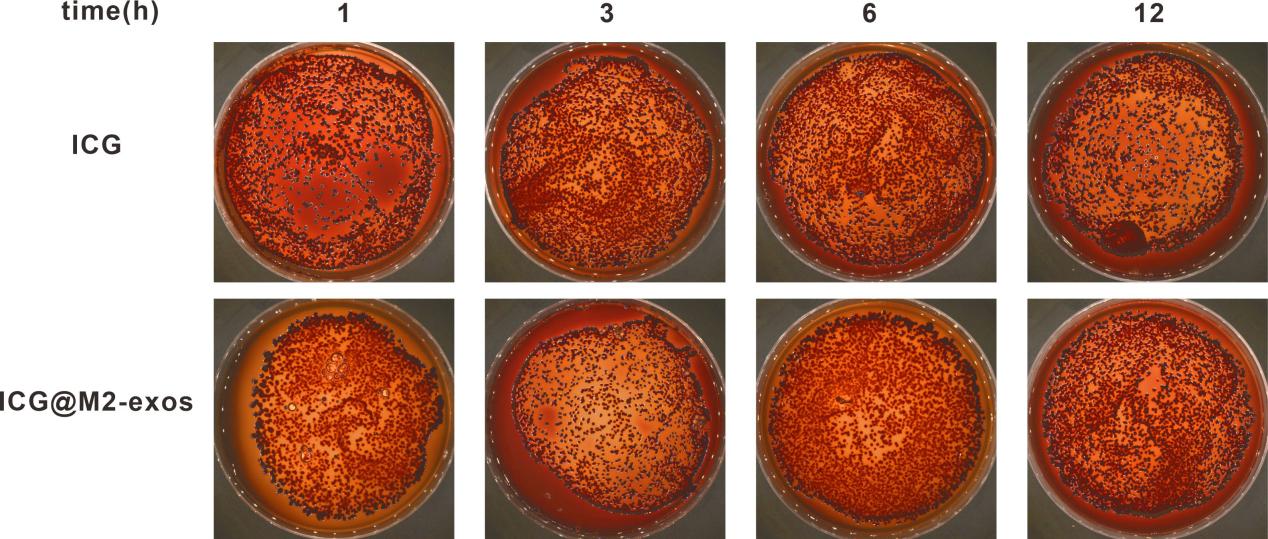


**Figure S1** The bacterial viability determined by plate spread assay (Control group images).

**Table S2**

| **Group** | **Time (hour)** | **CFU counting** | **Dilution factor** | **CFU/mL** | **Measure average diameter** |
| --- | --- | --- | --- | --- | --- |
| ICG | 1 | 556 | 0.00001 | 556000000 | 0.57 |
| ICG | 3 | 523 | 0.00001 | 523000000 | 0.78 |
| ICG | 6 | 558 | 0.00001 | 558000000 | 0.79 |
| ICG | 12 | 657 | 0.00001 | 657000000 | 0.92 |
| ICG@M2-exos | 1 | 567 | 0.00001 | 567000000 | 0.67 |
| ICG@M2-exos | 3 | 577 | 0.00001 | 577000000 | 0.79 |
| ICG@M2-exos | 6 | 598 | 0.00001 | 598000000 | 0.79 |
| ICG@M2-exos | 12 | 623 | 0.00001 | 623000000 | 0.82 |

Dynamics of bacterial concentration and colony morphology over time. Changes in CFU/mL and the corresponding average colony diameter during 12 h of pre contact culture under various treatment (ICG and ICG@M2-exos).

**Table S3**

| **Group** | **Irradiation Time (second)** | **CFU counting** | **Dilution factor** | **CFU/mL** | **Measure average diameter** |
| --- | --- | --- | --- | --- | --- |
| ICG | 0 | 961 | 0.00001 | 961000000 | 1.12 |
| ICG | 60 | 375 | 0.00001 | 375000000 | 1.31 |
| ICG | 120 | 131 | 0.00001 | 131000000 | 1.21 |
| ICG | 180 | 74 | 0.00001 | 74000000 | 1.27 |
| ICG@M2-exos | 0 | 920 | 0.00001 | 920000000 | 1.06 |
| ICG@M2-exos | 60 | 455 | 0.00001 | 455000000 | 1.17 |
| ICG@M2-exos | 120 | 236 | 0.00001 | 236000000 | 1.24 |
| ICG@M2-exos | 180 | 112 | 0.00001 | 112000000 | 1.15 |

Dynamics of bacterial concentration and colony morphology over time. Changes in CFU/mL and the corresponding average colony diameter during 180 s of 808 nm laser irradiation under various treatment (ICG and ICG@M2-exos).


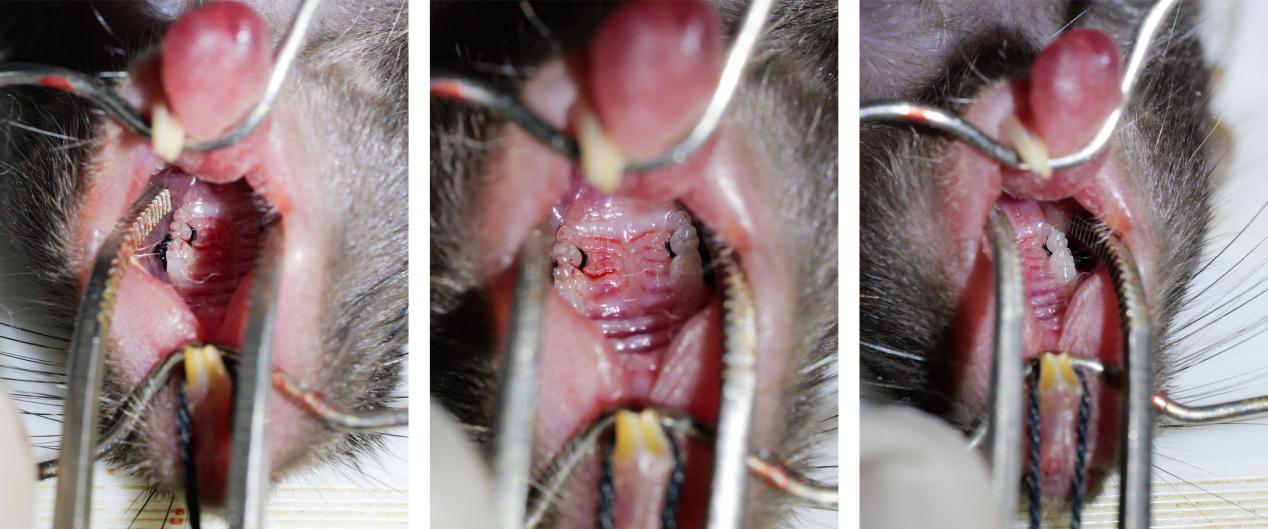


**Figure S2** Key steps in the physical model assembly process (Periodontitis was induced in Sprague-Dawley rats over 3 weeks: maxillary second molar gingival sulci were ligated with silk sutures for 2 h to facilitate bacterial invasion, followed by antibiotic suppression of native flora).


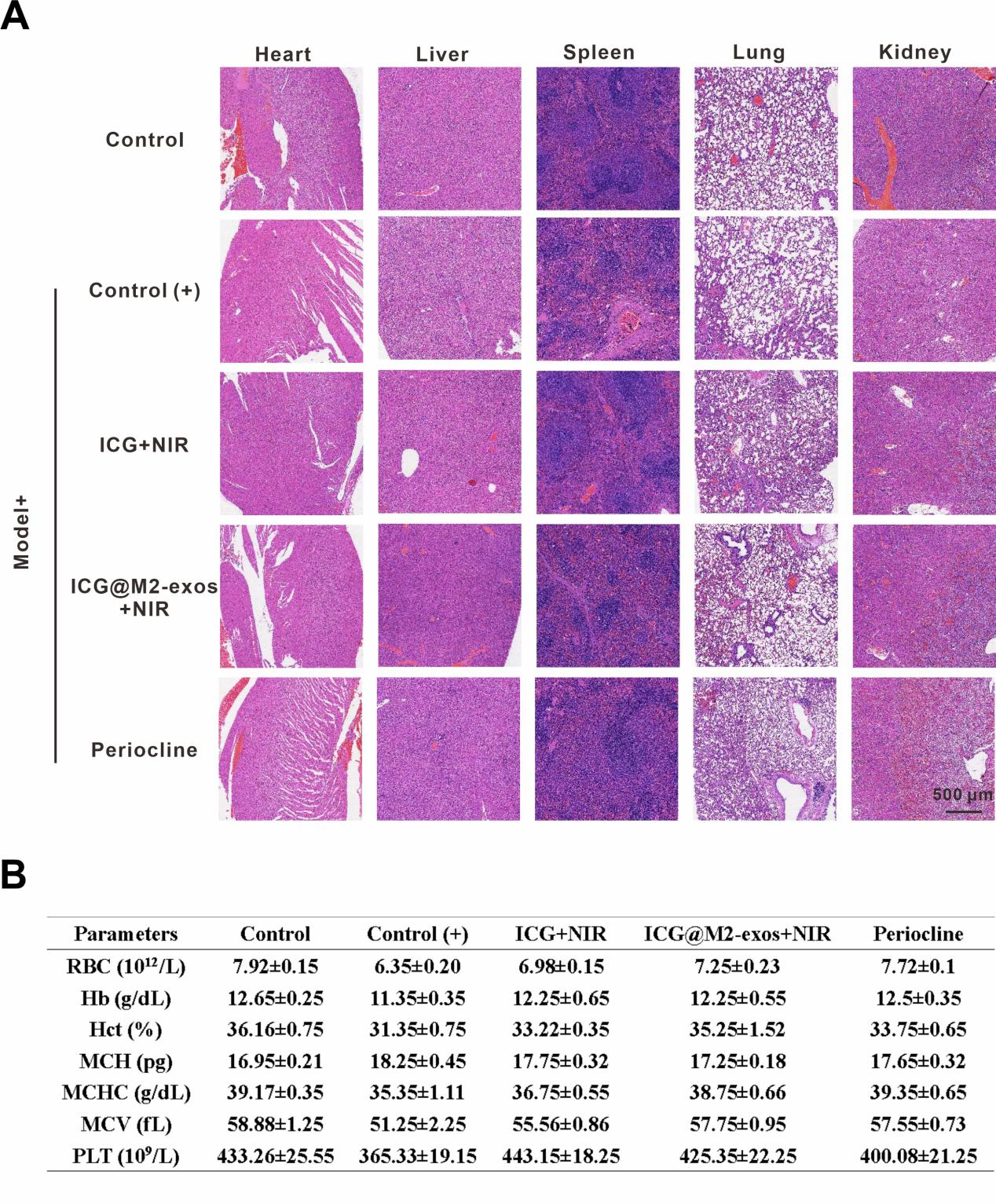


**Figure S3** *In vivo* biosafety evaluation of the treatment. (A) Representative H&E-stained images of major organs (heart, liver, spleen, lungs, and kidneys) collected at the endpoint of the study. No significant histological abnormalities or pathological lesions were observed across all experimental groups. (B) Hematological toxicity analysis of blood samples from each group. Key parameters, including liver and kidney function markers, showed no notable differences, indicating the absence of systemic toxicity.
