# Supplementary figures and images for: NIR-Activated ICG-Loaded M2 Macrophage Exosomes Ameliorate Periodontitis via Targeting Infection Inflammation and Oxidative Stress
Source: Research (Wash D C). 2026 Apr 27;9:1207. doi: 10.34133/research.1207 (PMC13113311; doi:10.34133/research.1207)

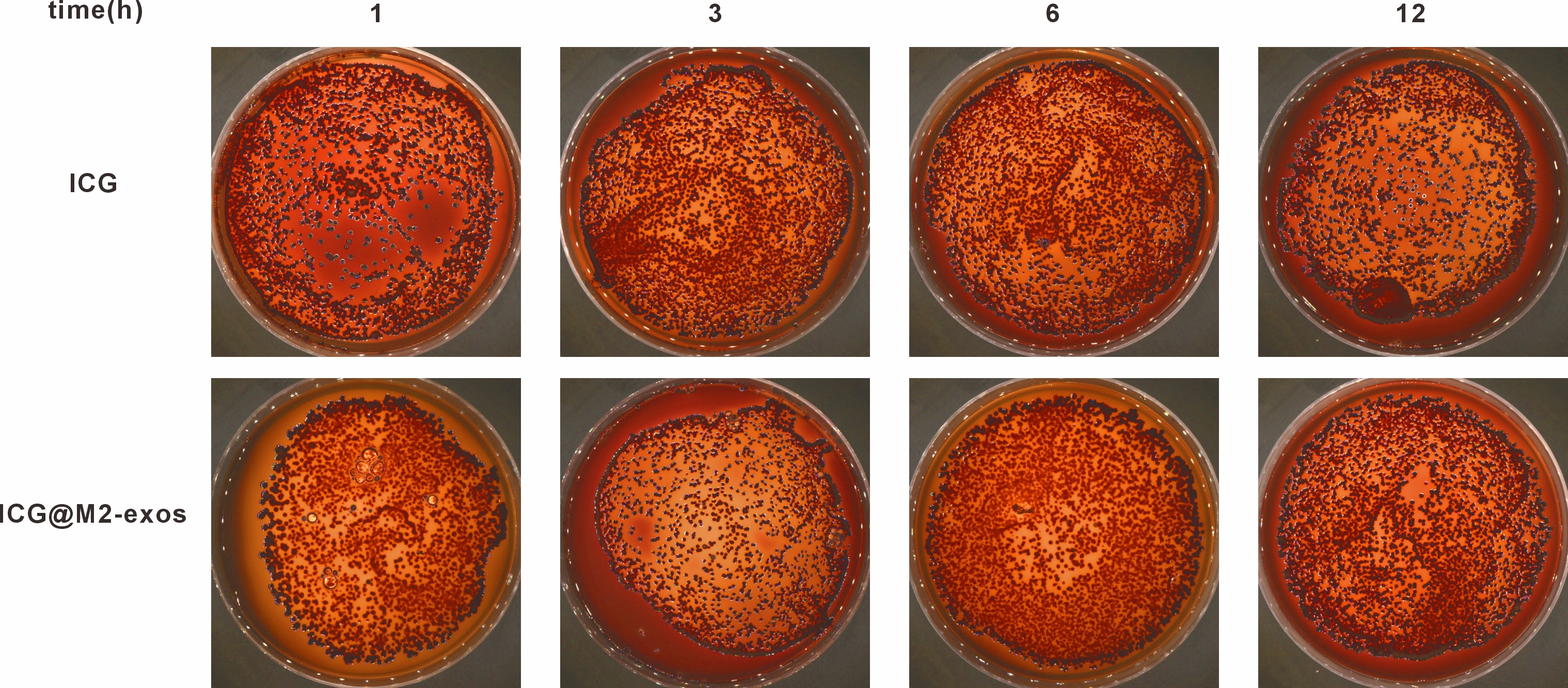

Supplement: Supplementary 1 — Figs. S1 to S4 Tables S1 to S4 [file research.1207.f1.zip › Fig S1.jpg]

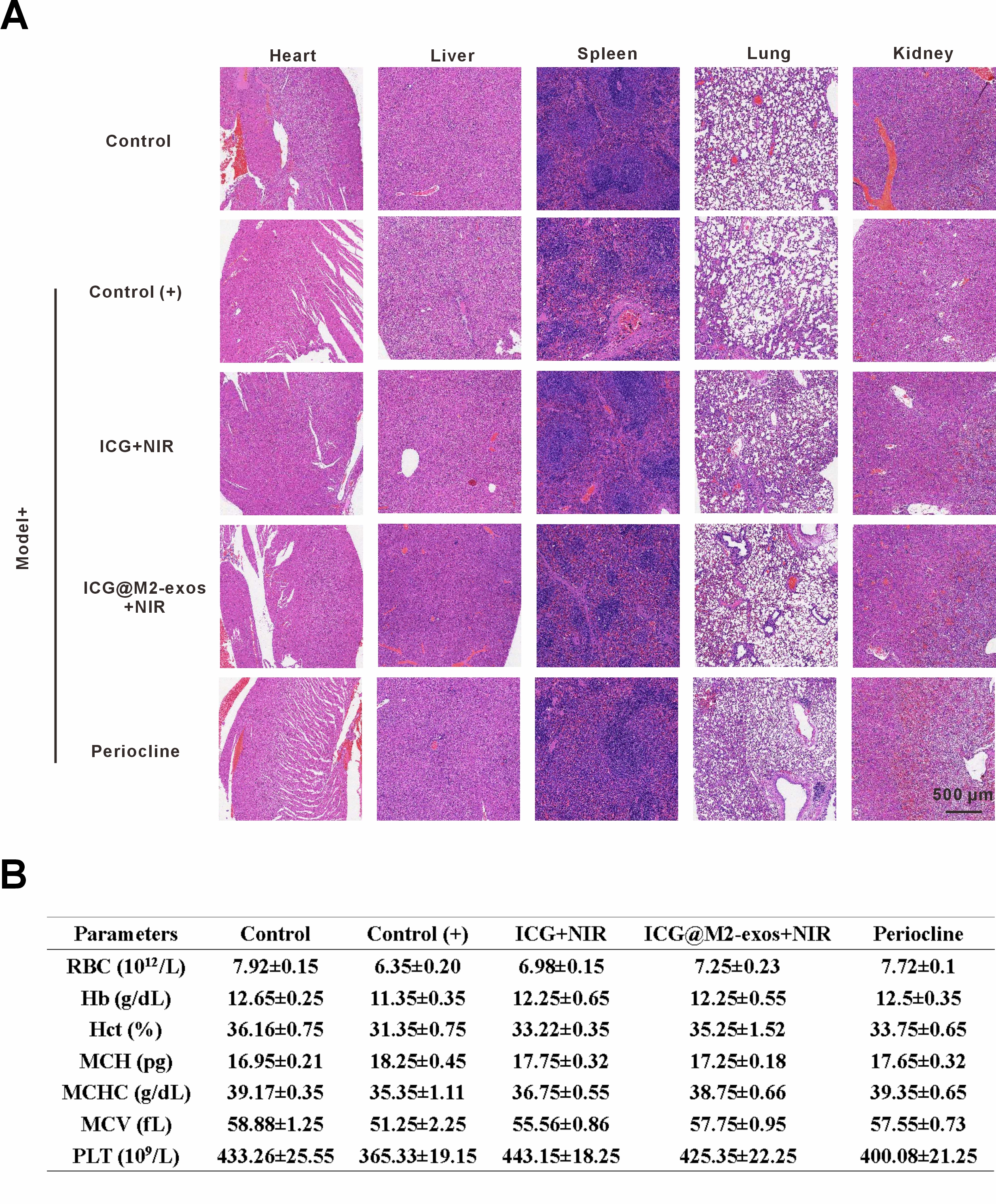

Supplement: Supplementary 1 — Figs. S1 to S4 Tables S1 to S4 [file research.1207.f1.zip › Fig S3.jpg]

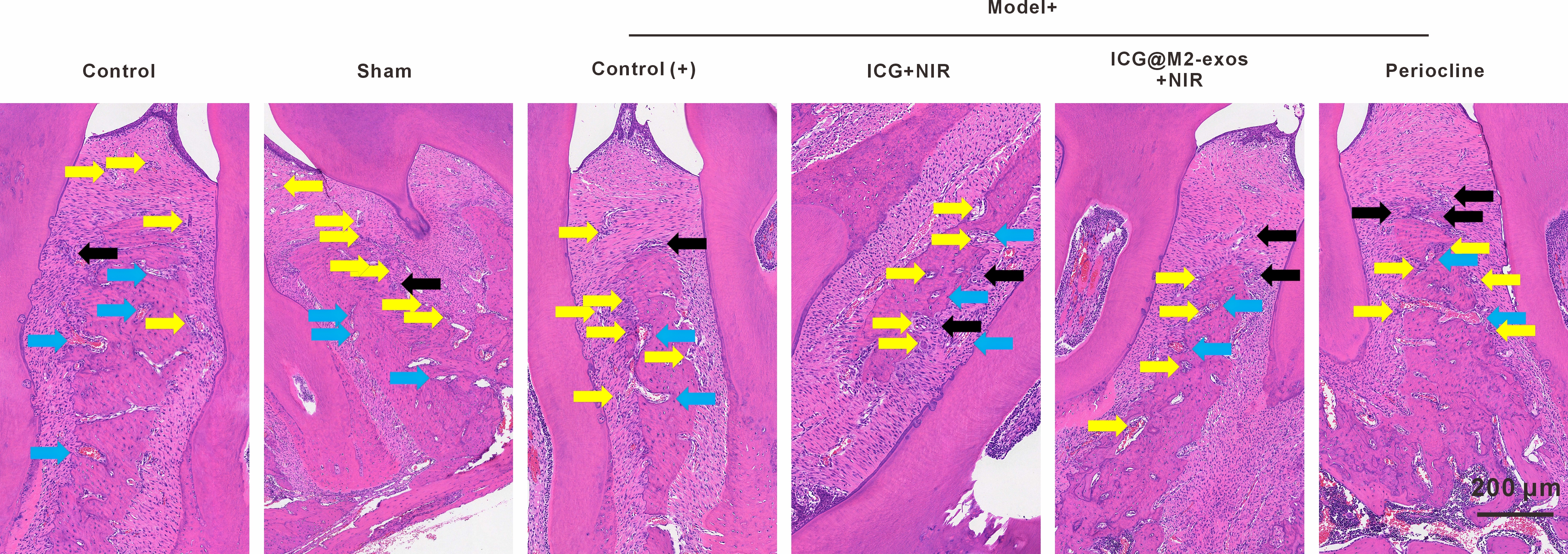

Supplement: Supplementary 1 — Figs. S1 to S4 Tables S1 to S4 [file research.1207.f1.zip › Fig S4.jpg]

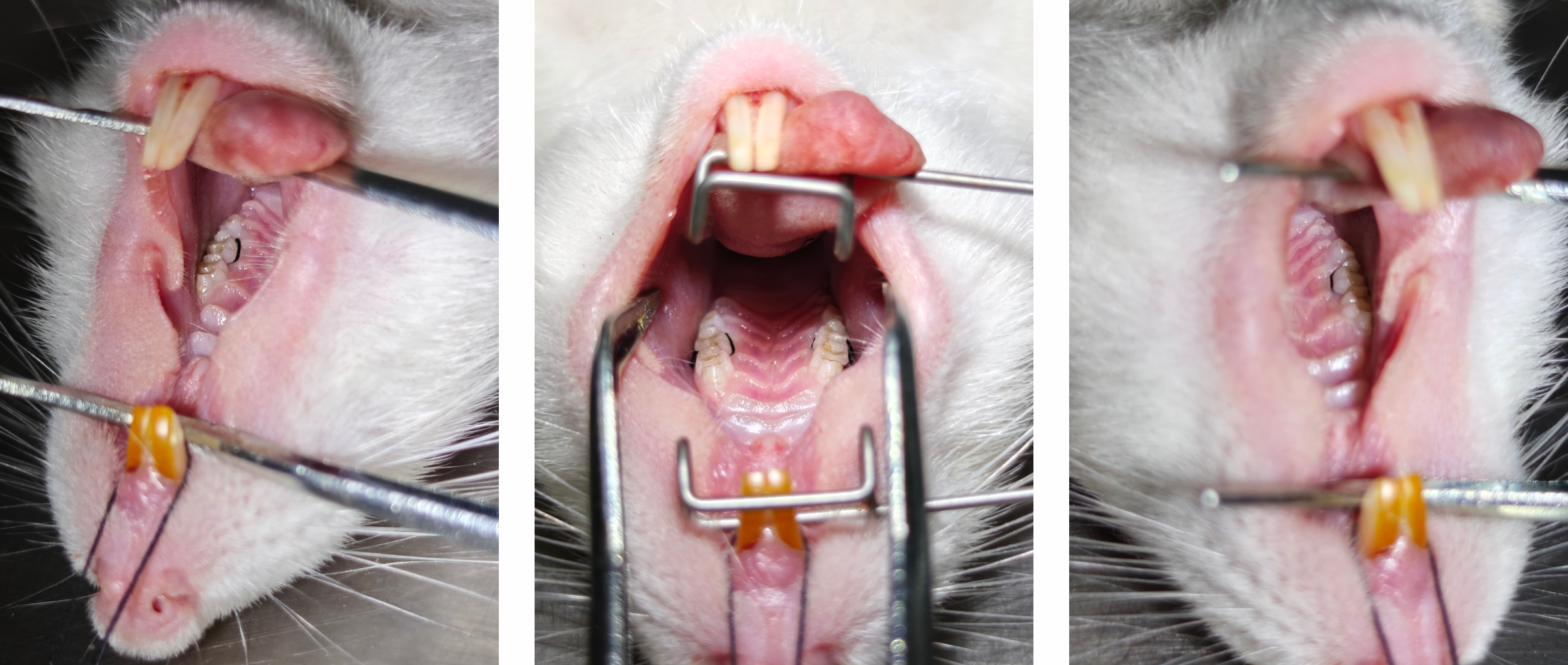

Supplement: Supplementary 1 — Figs. S1 to S4 Tables S1 to S4 [file research.1207.f1.zip › Figure S2-SD_rat_corrected.jpg]
